# Impact along the HIV pre‐exposure prophylaxis “cascade of prevention” in western Kenya: a mathematical modelling study

**DOI:** 10.1002/jia2.25527

**Published:** 2020-06-30

**Authors:** Anna Bershteyn, Monisha Sharma, Adam N Akullian, Kathryn Peebles, Supriya Sarkar, R Scott Braithwaite, Edinah Mudimu

**Affiliations:** ^1^ Department of Population Health NYU Grossman School of Medicine New York NY USA; ^2^ Institute for Disease Modeling Bellevue WA USA; ^3^ Department of Global Health University of Washington Seattle WA USA; ^4^ Department of Epidemiology Emory University Atlanta GA USA; ^5^ Department of Epidemiology University of Washington Seattle WA USA; ^6^ Department of Decision Sciences University of South Africa Pretoria South Africa

**Keywords:** HIV prevention, pre‐exposure prophylaxis, cascade, mathematical modelling, Kenya, sub‐Saharan Africa

## Abstract

**Introduction:**

Over one hundred implementation studies of HIV pre‐exposure prophylaxis (PrEP) are completed, underway or planned. We synthesized evidence from these studies to inform mathematical modelling of the prevention cascade for oral and long‐acting PrEP in the setting of western Kenya, one of the world’s most heavily HIV‐affected regions.

**Methods:**

We incorporated steps of the PrEP prevention cascade – uptake, adherence, retention and re‐engagement after discontinuation – into EMOD‐HIV, an open‐source transmission model calibrated to the demography and HIV epidemic patterns of western Kenya. Early PrEP implementation research from East Africa was used to parameterize prevention cascades for oral PrEP as currently implemented, delivery innovations for oral PrEP, and future long‐acting PrEP. We compared infections averted by PrEP at the population level for different cascade assumptions and sub‐populations on PrEP. Analyses were conducted over the 2020 to 2040 time horizon, with additional sensitivity analyses for the time horizon of analysis and the time when long‐acting PrEP becomes available.

**Results:**

The maximum impact of oral PrEP diminished by over 98% across all prevention cascades, with the exception of long‐acting PrEP under optimistic assumptions about uptake and re‐engagement after discontinuation. Long‐acting PrEP had the highest population‐level impact, even after accounting for possible delays in product availability, primarily because its effectiveness does not depend on drug adherence. Retention was the most significant cascade step reducing the potential impact of long‐acting PrEP. These results were robust to assumptions about the sub‐populations receiving PrEP, but were highly influenced by assumptions about re‐initiation of PrEP after discontinuation, about which evidence was sparse.

**Conclusions:**

Implementation challenges along the prevention cascade compound to diminish the population‐level impact of oral PrEP. Long‐acting PrEP is expected to be less impacted by user uptake and adherence, but it is instead dependent on product availability in the short term and retention in the long term. To maximize the impact of long‐acting PrEP, ensuring timely product approval and rollout is critical. Research is needed on strategies to improve retention and patterns of PrEP re‐initiation.

## 1 INTRODUCTION

HIV is a leading cause of death in sub‐Saharan Africa [1], particularly in Eastern and Southern Africa, which contain a majority of the world’s HIV burden and incidence [2]. Western Kenya contains some of the world’s hardest‐hit communities, with approximately one in four adults living with HIV in the counties of Homa Bay and Siaya [3]. Over the past decade, rates of new HIV infections have declined in western Kenya, concurrently with scale‐up of HIV treatment and preventative interventions such as male circumcision, but remain among the highest rates observed globally [4].

Oral pre‐exposure prophylaxis (PrEP) reduces the risk of HIV acquisition [5, 6], but implementation has been slow to expand in Eastern and Southern Africa [7] due to numerous social, political, cultural and logistical barriers [8]. Some of these barriers have a multiplicative effect, compounding to make population‐level impact more challenging.

A “prevention cascade” framework has been proposed for HIV [9] and for PrEP in particular [10, 11] to organize the components of PrEP implementation that have an effect on HIV prevention. This is analogous to the HIV “treatment cascade,” or care continuum framework, widely adopted for treatment of people living with HIV (PLHIV), which helped to inform the UNAIDS 90‐90‐90 targets for HIV diagnosis, treatment and viral load suppression [12]. Here we adapt a previously developed agent‐based HIV network transmission model, which includes a detailed model of HIV care and prevention, to simulate the cascades of prevention affecting the population‐level impact of PrEP. Our analysis provides quantitative insights into a relatively new and increasingly critical concept in HIV prevention research.

## 2 Methods

### 2.1 Model of HIV in western Kenya

We developed a cascade of prevention model for PrEP within the health care component of EMOD‐HIV, part of the EMOD transmission modelling software tool [13, 14]. EMOD‐HIV is an HIV epidemiological model that integrates population demography, HIV disease progression; and network‐based HIV transmission configured to match age‐ and sex‐specific propensities to form different sexual partnership types [15]. Interventions such as antiretroviral therapy (ART), voluntary medical male circumcision (VMMC), and PrEP are added to EMOD through a highly configurable health care module in which different steps of health seeking and outreach can be targeted to sub‐populations and can be made to vary over time [16].

Prior to the current analysis, EMOD had been calibrated to fit the HIV epidemic in six counties in western Kenya: Siaya, Kisumu, Homa Bay, Migori, Kisii and Nyamira [17]. Model calibration methods, parameter values and their distributions, and quality of fit to HIV survey and routine data, have been published in detail elsewhere [17] and are summarized below, with emphasis on those model components most closely related to HIV prevention. Because PrEP rollout did not begin until 2017 – whereas the most recently available HIV prevalence survey was conducted in 2012 – the pre‐existing model was not re‐calibrated. As a model validation exercise, EMOD‐HIV was calibrated to demographic, HIV prevalence, and viral suppression data from 32 high‐incidence communities in Eastern Africa, including 16 communities in western Kenya, that were enrolled in a community‐randomized trial of ART scale‐up [18]. The model successfully predicted HIV incidence in these communities while still blinded to measured incidence, the trial’s primary outcome [19].

Both traditional male circumcision and scale‐up of VMMC were included using age‐ and county‐specific circumcision coverage estimates from the Demographic and Health Surveys (DHS) [20]. Importantly, the model incorporated county‐specific estimates of the population sizes of commercial sex workers based on an enumeration conducted in 2012, the same year as the most recently available HIV prevalence estimate [21].

The calibration target data, model parameters and fitted modelled trajectories for the six‐county western Kenya model have been described in detail [17]. Briefly, the agent‐based model represented the western Kenyan population, with an *in silico* model population equal to one‐25th the population of western Kenya. The population structure was fit to match the Kenya Population and Housing Census, and with age‐specific fertility rates and age‐ and sex‐specific non‐HIV mortality rates published by the UN Population Division [22, 23]. The model was fit to age‐, sex‐ and county‐stratified HIV prevalence estimates from 2003 and 2008 to 2009 DHS Surveys, 2007 and 2012 AIDS Indicator Surveys, numbers of PLHIV on ART reported annually by the Kenya Ministry of Health, and population statistics from the Kenya Population and Housing Census [24, 25].

Modelling fitting to data was performed using parallel simultaneous perturbation optimization, an algorithm that maximizes the posterior probability of the model’s fit to data [26, 27]. The cost function being minimized in the calibration process was the log‐likelihood of the model’s fit to population age/sex structure, number on ART, and age‐, sex‐ and country‐stratified HIV prevalence after accounting for the uncertainty of each estimate based on the survey design and sample size. After model fitting, 250 trajectories were obtained by resampling parameter sets among all those run during the fitting process in proportion to the likelihood score of each simulation. To estimate the impact of only historical levels of PrEP, for which the total number of averted infections is still small, the number of replicates was increased to 2000 model runs.

### 2.2 Baseline levels of PrEP usage

County‐specific estimates of PrEP use since the start of rollout were obtained from the U.S. President’s Emergency Plan for AIDS Relief (PEPFAR) [28], and national‐level estimates were obtained from PrEPWatch [29]. PrEPWatch estimated the total number of PrEP users in Kenya in 2019 to be 44,000. Among the 14,258 PrEP users included in the PEPFAR dataset, 7866 resided in six counties comprising the former Nyanza province of western Kenya. We used the proportion of users in each county relative to the total to allocate the PrEPWatch estimates across the six counties of the Nyanza region, yielding a total of 24,274 users within Nyanza. The baseline scenario allocations by county were: 8,243 (34%) in Siaya, 5120 (21%) in Kisumu, 4820 (20%) in Homa Bay; 4663 (19%) in Migori; 1102 (5%) in Kisii; and 327 (1%) in Nyamira. To model continuation of 2019 levels of PrEP use, we simulated 25,000 users annually with the same proportions by county: 8489 in Siaya; 5273 in Kisumu; 4964 in Homa Bay; 4802 in Migori; 1135 in Kisii; and 337 in Nyamira.

### 2.3 Target populations for PrEP scale‐up

Scenarios of PrEP rollout were modelled by increasing the numbers of individuals on PrEP in four target populations: (1) adolescents and adults ages 15 to 29 living in areas with HIV prevalence exceeding 10%, which for western Kenya were Homa Bay, Siaya, Kisumu and Migori Counties; (2) adolescent girls and young women (AGYW) ages 15 to 24 living in counties with HIV prevalence exceeding 10%; (3) higher‐risk AGYW, defined as female sex workers and women likely to have multiple sex partners in all counties, (4) and higher‐risk males, defined as males in the high‐incidence age group of 20 to 29 years who are clients of sex workers or likely to have multiple sex partners in all counties. Risk of multiple sex partners was modelled as a propensity to acquire multiple simultaneous sex partners within the sexual network – with partner acquisition rates varying by age according to the matrix of age‐dependent assortativity – as well as a three‐fold higher risk of co‐infection with a sexually transmitted infection.

### 2.4 PrEP cascade steps

**Figure 1:**
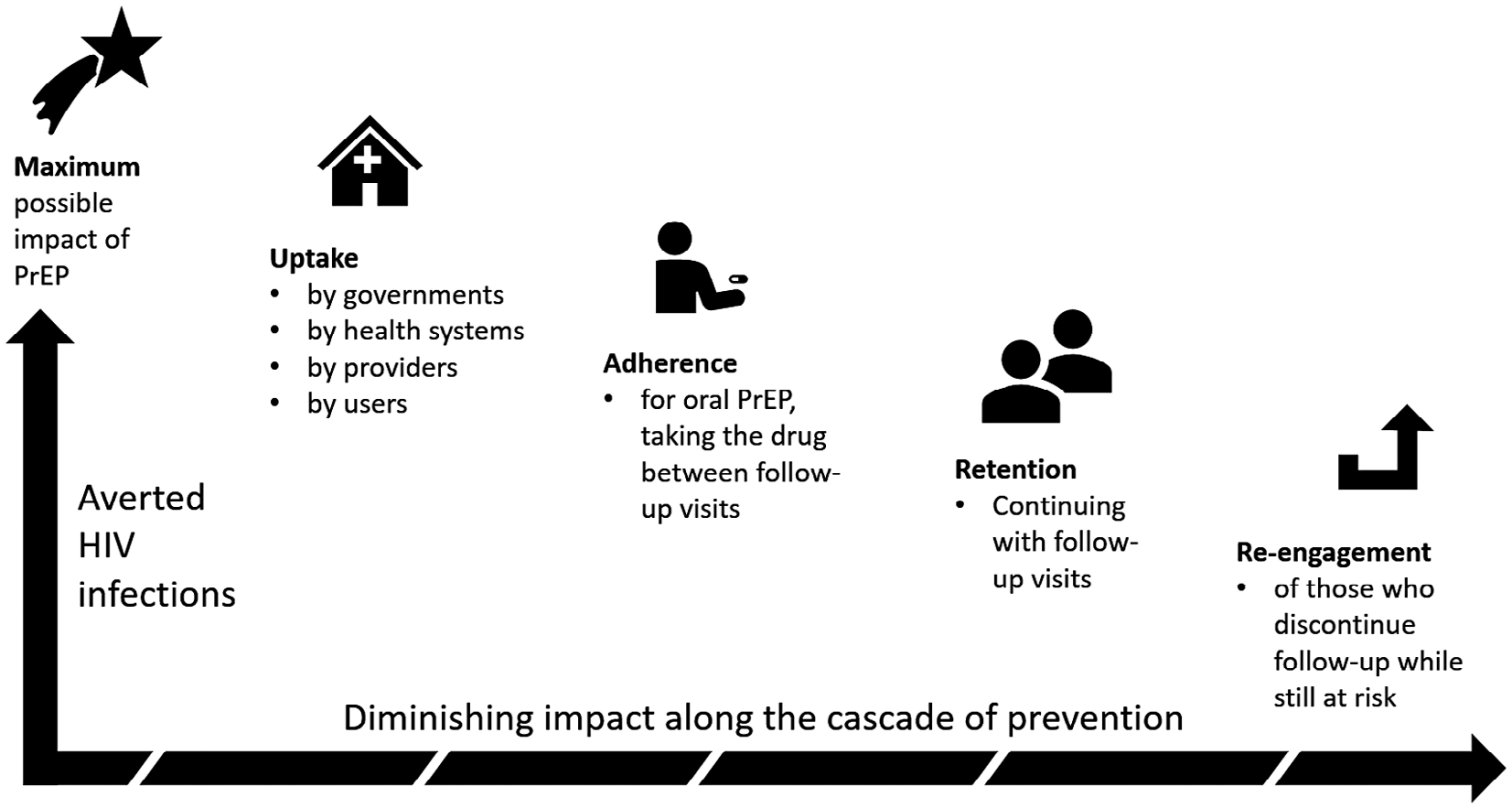
Diagram of the prevention cascade added to the EMOD‐HIV model. PrEP, pre‐exposure prophylaxis

Previously published conceptual frameworks of the prevention cascade [9, 10, 11] have considered numerous potential steps of the HIV prevention cascade. To simplify the analysis given the paucity of data on individual steps, our analysis consolidates the proposed frameworks into four steps that they share in common: uptake, adherence, retention, and re‐engagement (Figure 1). To parameterize each step, we reviewed current and ongoing PrEP implementation and demonstration projects referenced in review articles [8, 30, 31, 32] and tabulated by the AIDS Vaccine Advocacy Coalition [33]. Of the 108 PrEP studies globally, 43 had at least one site in sub‐Saharan Africa and 15 were completed as of April 2019. Published results from these studies were used to inform composite model assumptions of three potential prevention cascades: (1) currently available oral PrEP implementation (“oral conventional”), (2) future innovation in PrEP implementation strategies (“oral innovative”), and (3) new long‐acting PrEP products (“long‐acting”).

### 2.5 PrEP cascade parameterization

Assumptions regarding each step of the three PrEP cascades are summarized in Table 1. To represent the PrEP cascade achievable with currently available methods of oral PrEP delivery (“oral conventional”), we drew on existing data and coverage targets. For uptake, we used the World Health Organization (WHO) target of 10% coverage by 2020 of high‐risk populations, defined as young people in high‐prevalence settings and key populations in all settings [34]. This target is consistent with the achieved coverage in PrEP scale‐up projects in sub‐Saharan Africa [35, 36]. Uptake in the model is defined as the proportion of individuals initiating PrEP annually.

**Table 1:** Prevention cascade model scenarios, assumptions and ranges used in sensitivity analysis

Cascade	Step	Assumption	Sources
Conventional oral PrEP	Uptake	10%	[34, 35, 36]
	Adherence	36% to 75%	[5, 37]
	Retention	3 months	[35, 38, 39]
	Reengagement	0% to 100%	No available data
Innovative delivery of oral PrEP	Uptake	30%	[38]
	Adherence	61% to 75%	[5, 37]
	Retention	9 months	[35, 38, 39]
	Reengagement	0% to 100%	No available data
Long‐acting PrEP	Uptake	10% to 50%	[40]
	Adherence	95%	[40]
	Retention	9 months	[35, 38, 39]
	Reengagement	0% to 100%	No available data

PrEP, pre‐exposure prophylaxis.

To represent how ongoing oral implementation research can improve the PrEP cascade (“oral innovative”), we used results from a study conducted in Uganda which found that both uptake and retention on PrEP were three‐fold higher among users who resided within two kilometres of a PrEP‐dispensing clinic, after adjusting for other factors [38]. We assumed that innovations to overcome barriers to PrEP uptake and continuation could raise uptake three‐fold to 30%. Our optimistic assumptions about long‐acting PrEP assumed even higher uptake of 50% based on user preference research in western Kenya [40], while pessimistic assumptions assumed an uptake of 10%, the same as the WHO target for conventional oral PrEP.

Adherence patterns for daily oral PrEP are highly variable and have a strong correlation with PrEP efficacy. A meta‐analysis of five clinical trials of oral PrEP in women in estimated an efficacy of 36% across all study participants, and 61% for participants with high adherence (defined as having detectable levels of PrEP drugs in 75% of blood samples) [37]. In the western Kenya setting, a study estimated far higher PrEP efficacy of 75% [5]. Therefore, we examined as pessimistic assumptions an adherence‐driven efficacy of 36% for conventional PrEP and 61% for PrEP with innovative implementation, and an optimistic assumption of 75% efficacy for both conventional and innovative PrEP implementation. Since long‐acting PrEP is independent of user adherence to daily pills (but rather reliant on continued clinical care) we assumed efficacy for long‐acting PrEP was 95%, the same level as the upper limit scenario.

Retention has been a significant challenge in PrEP implementation. Among AGYW ages 15 to 29 initiating PrEP in Kenya, only 5% remained on PrEP after ten months [39]. In a large prevention project in Kenya and Uganda, the average duration of PrEP use was three months [35, 38]. Therefore, we assumed a three‐month retention period on PrEP. Drawing on the study of PrEP users living close to PrEP‐dispensing clinics, we assumed that the mean duration on PrEP could be tripled, from three months to nine months, through innovative implementation [38].

We did not find evidence to inform the fourth step of the prevention cascade: re‐engagement of clients who have discontinued PrEP. Therefore, two model scenarios were compared: one in which users who discontinued PrEP would not re‐initiate PrEP, and one in which PrEP re‐initiation after discontinuation occurs at the same rate as for other PrEP‐eligible individuals.

For each cascade step, the attenuation in PrEP impact was estimated as number of infections averted at the population level – including among those not on PrEP – relative to a baseline scenario in which PrEP usage increased at a steady rate based on 2019 levels. In lieu of annual discounting of impact, which is commonly performed in cost‐effectiveness analyses, we instead used more interpretable undiscounted time horizons of 5, 10, 20 and 30 years ending in 2025, 2030, 2040 and 2050 respectively with all analyses beginning in 2020.

### 2.6 Timelines for PrEP rollout

For oral innovative PrEP, we assumed that optimized strategies for PrEP delivery would be identified by 2021, based on projected completion dates of ongoing implementation studies. To account for the time required to implement new evidence‐based strategies, we assumed innovative PrEP rollout ramped up in a linear fashion from 2021 to 2023. For long‐acting PrEP, an ongoing clinical trial to measure efficacy among women in sub‐Saharan Africa has an estimated completion date of May 2022 [41]. We assumed the most optimistic rollout would begin in 2023 with a two‐year ramp up until 2025. This rapid timeline for long‐acting PrEP was taken as an upper limit, and we compared how impact might diminish if long‐acting PrEP availability were delayed until 2025, 2027 or 2029.

## 3 RESULTS

**Figure 2:**
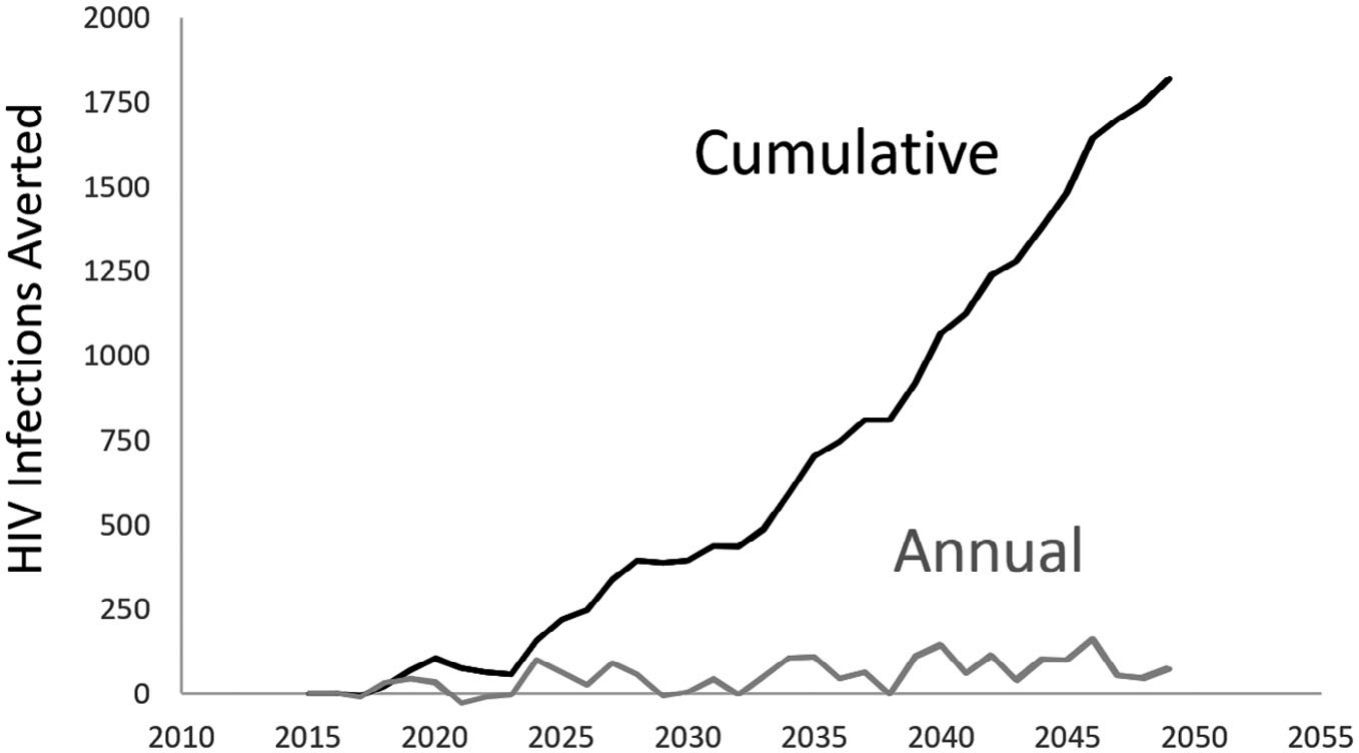
Infections averted by oral PrEP used at current levels in western Kenya. PrEP use was simulated for the six counties of Nyanza using estimates from PrEPWatch, and stratified at the county level using data from PEPFAR. As a conservative assumption, this analysis assumed a continued rate of 25,000 annual PrEP users in Nyanza, just above the 24,274 users estimated for 2019. Impact is estimated at the population, so that estimates include averted infections in PrEP users as well as averted infections among individuals in the sexual network of PrEP users. Impact is modest because western Kenya is still early in the process of PrEP roll‐out. PrEP, pre‐exposure prophylaxis; PEPFAR: president's emergency plan for AIDS relief

The early PrEP rollout in western Kenya has already averted approximately 100 HIV infections annually, as shown in Figure 2. In a conservative projection that assumes no further expansion of PrEP beyond 2019 levels (approximately 25,000 users annually, each using PrEP for an average of three months), maintaining *status quo* PrEP availability could avert 2000 HIV infections in western Kenya by 2050.

**Figure 3:**
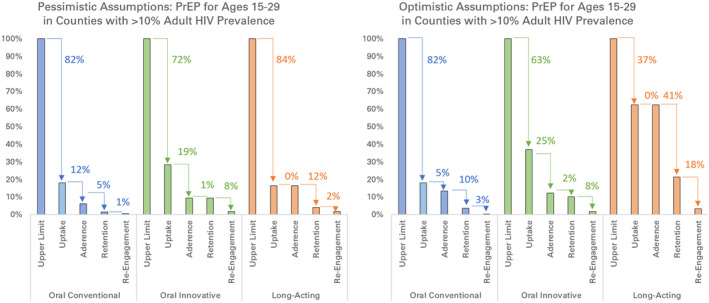
Percentage of infections averted along prevention cascades with pessimistic and optimistic assumptions. Arrows show absolute percentage decrease relative to the previous step of the cascade. Prevention cascades are shown for conventional implementation of oral PrEP (blue), innovative implementation of oral PrEP (green), and long‐acting PrEP (orange) in western Kenya, under assumptions described in Table 1. The population receiving PrEP is men and women ages 15 to 29 in counties with HIV prevalence exceeding 10% (Homa Bay, Siaya, Kisumu, and Migori). Infections averted are calculated for the entire population, including those not on PrEP. PrEP, pre‐exposure prophylaxis

Impact along the cascades diminished greatly due to compounding declines along each cascade step, under both pessimistic and optimistic assumptions about uptake and adherence (Figure 3). Although the model captured knock‐on effects of PrEP averting infections in the community, the overall prevention cascades were approximately proportional to the assumed reduction in uptake, adherence and annual retention, and dropped off considerably if those who previously disengaged from PrEP were unwilling to re‐initiate PrEP in the future.

Assuming those who discontinue PrEP could re‐initiate as often as annually, the end‐to‐end prevention cascades for conventional oral PrEP, innovative delivery of oral PrEP, and long‐acting PrEP had a drop‐off in infections averted of 98.5%, 90.4% and 95.9% respectively under pessimistic assumptions, and 96.5%, 89.8% and 78.6% respectively under optimistic assumptions. Assuming those who discontinue PrEP are unwilling to re‐initiate PrEP, the drop‐off in impact was >98% all cascades except for the long‐acting PrEP cascade under optimistic assumptions, where it was 96.6%.

The decline in impact along PrEP cascades was not sensitive to the target population receiving PrEP Figure S1 among the four populations tested: youth ages 15 to 29 in counties with >10% HIV prevalence, adolescent and young women ages 15 to 24 in counties with >10% HIV prevalence; sex workers and high‐risk women in all counties; or sex worker clients and higher‐risk men in all counties.

**Figure 4:**
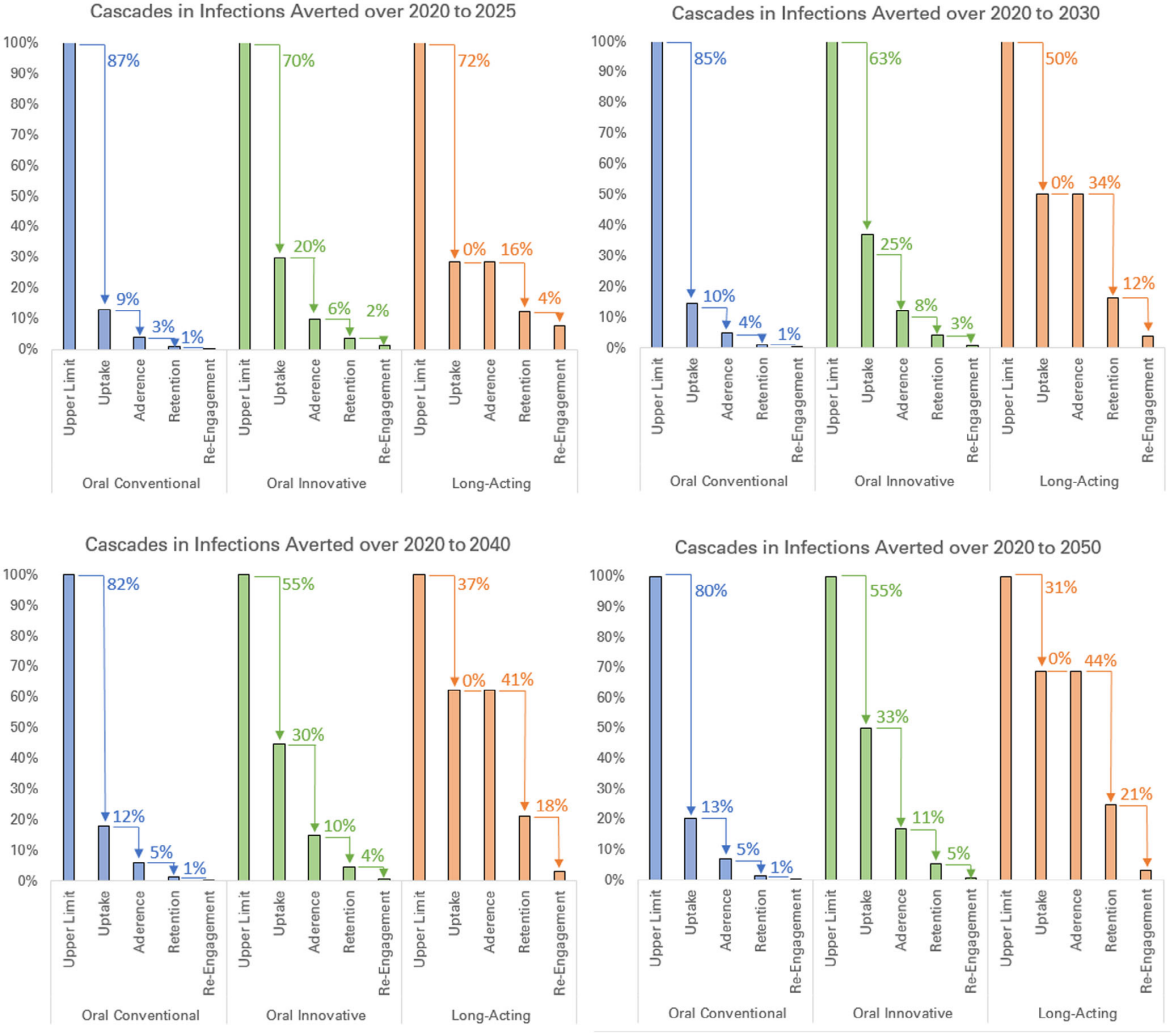
Percentage of infections averted along prevention cascades over different time horizons of analysis. Arrows show absolute percentage decrease relative to the previous step of the cascade. Prevention cascades for conventional implementation of oral PrEP (blue), innovative implementation of oral PrEP (green), and long‐acting PrEP (orange) in western Kenya were organized into the uptake, adherence, retention and re‐initiation of those at risk who have discontinued PrEP. The population receiving PrEP is men and women ages 15 to 29 in counties with HIV prevalence exceeding 10%. Infections averted is calculated for the entire population, including those not on PrEP. Infections averted are cumulative over time horizons of 2020 to 2025 (top left), 2020 to 2030 (top right), 2020 to 2040 (bottom left) and 2020 to 2050 (bottom right). PrEP, pre‐exposure prophylaxis

The time horizon of analysis substantially influenced the relative impact among the three prevention cascades Figure 4. In particular, optimism about the possible uptake of long‐acting PrEP was offset by the delay in its availability and time required to ramp up coverage, so that an optimistic long‐acting PrEP cascade was only distinguished itself from oral PrEP cascades over time horizons of ten or more years.

**Figure 5:**
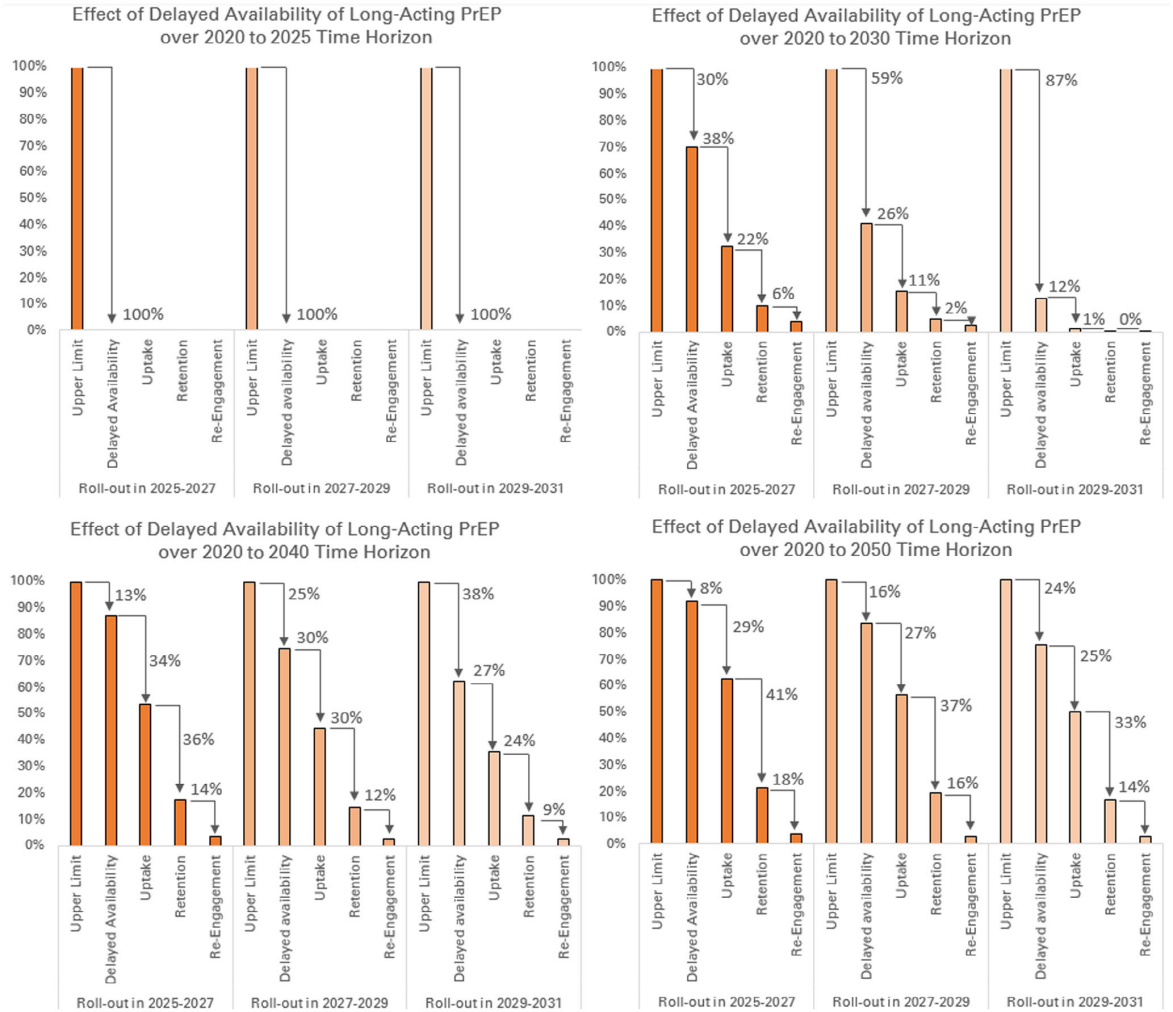
Percentage of infections averted along prevention cascades for long‐acting PrEP for different timelines of product rollout and different time horizons of analysis. Adherence is not included in the depicted cascades; return for injections is subsumed into the retention step of the cascade. The target group is adults 15 to 29 in counties with prevalence above 10%. Infections averted are cumulative over time horizons of 2020 to 2025 (top left), 2020 to 2030 (top right), 2020 to 2040 (bottom left) and 2020 to 2050 (bottom right). PrEP, pre‐exposure prophylaxis

The timeline for rollout of long‐acting PrEP in sub‐Saharan Africa is uncertain. We examined how delayed rollout, relative to the optimistic timeline of a 2023 launch with a linear ramp‐up through 2025, would diminish the impact of long‐acting PrEP under optimistic assumptions (Figure 5). As expected, a product launch later than 2025 provides no opportunity for impact over the 2020 to 2025 period and would greatly diminish impact over a 10‐year time horizon. However, examined over longer time horizons of 20 or 30 years, long‐acting PrEP would out‐perform oral PrEP under optimistic assumptions.

## 4 DISCUSSION

Analogous to the care cascade for HIV treatment, we examined the use of an HIV prevention cascade to evaluate how different steps compound to influence population‐level impact of PrEP in terms of HIV infections averted. We found that the population‐level impact of PrEP diminishes considerably across the cascade of prevention. For oral PrEP, the largest drop‐off in PrEP impact occurred from limited uptake. Even with optimistic assumptions about the impact of implementation innovations, uptake remained the greatest drop‐off in the oral PrEP cascade. For long‐acting PrEP, it is not known whether uptake will be limited to levels similar to that of oral PrEP as the main limiting factor. Under optimistic assumptions about uptake, the main limiting factor for long‐acting PrEP would be retention.

With the exception of long‐acting PrEP under optimistic assumptions about uptake, all other cascades caused a diminishing of >98% of impact after aggregating the compounding effects of each step of the cascades. Long‐acting PrEP, under optimistic assumptions about uptake and re‐engagement, still lost a substantial 78.6% of its impact long the cascade, highlighting the formidable challenge of compounding steps in prevention cascades even in the best of circumstances.

Introduction of long‐acting PrEP could enable a shift of resources from adherence‐focused interventions to ones that maximize retention and re‐engagement. Such interventions might address support from intimate partners, perception of HIV risk, reducing and coping with stigma, and prioritization of PrEP among competing priorities of providers and users [42, 43]. In addition, the impact of long‐acting PrEP was highly sensitive to assumptions about re‐engagement after PrEP interruption. The current evidence gap about willingness to re‐engage in PrEP will be especially critical to address for long‐acting PrEP.

Our study has several important limitations. Evidence about the cascades of prevention, especially the “oral innovative” and “long‐acting” PrEP cascades that have not yet been implemented, is limited to extrapolation from studies of oral conventional PrEP. We encountered the greatest evidence gaps for the final step of re‐engagement of those who have discontinued PrEP. In light of uncertainty, we modelled several possible long‐acting PrEP launch dates (2023, 2025, 2027 and 2029). Although 2029 is likely pessimistic since oral PrEP has improved readiness to adopt long‐acting PrEP, it is possible that unexpected challenges could arise from trial results, regulatory approval, supply or other prerequisites to the launch of long‐acting PrEP in Africa. Our analysis should be revisited as new information becomes available regarding the likely timeline of long‐acting PrEP rollout.

Owing to the paucity of data on prevention cascades, our analysis consolidated more detailed frameworks for prevention cascades into just a small number of cascade steps that those frameworks share in common. Expanding evidence about HIV prevention cascades may allow future analyses to disaggregate steps of the cascade that have been consolidated in this analysis. Future analyses could also leverage findings about integration of PrEP into existing HIV and non‐HIV services, for example, whether PrEP availability influences rates of HIV testing, thus conferring additional benefits by supporting prompt HIV diagnosis.

There is a lack of empiric data on the overlap between HIV risk and the cascade of prevention, especially in terms of risk‐driven patterns of discontinuation and re‐engagement. Given evidence of logistical barriers to oral conventional PrEP retention and adherence, we conservatively assumed that adherence, retention, and re‐engagement occurred independently of time periods of risky behaviour. Qualitative research suggests that users have intentions to discontinue PrEP when HIV risk is lower, and re‐engage when risk is higher [44]. There is a negative association between PrEP use and psychosocial factors such as stigma and depression, which may increase HIV risk and reduce protective behaviours other than PrEP use [45]. To the extent that PrEP engagement may have a net positive correlation with HIV risk, our conservative assumptions overestimate the fall‐off in impact across the prevention cascade.

Although our analysis attempts to leverage early results from PrEP implementation studies, a majority of global PrEP implementation studies are still ongoing. In addition, current users of PrEP may be biased toward “early adopters” and may not be representative of the population targeted to receive PrEP. Our analysis may necessitate revisiting if new future implementation science exposes different PrEP cascade parameters compared to early evidence.

Finally, our model used simple demographic criteria (age, sex and geographic location) along with general stratification of HIV risk (sex work, multiple sex partners and STI co‐infection rates) to determine the populations targeted for PrEP. Future, more detailed modelling should be coupled with emerging evidence on the characteristics of those to whom PrEP is offered at scale. Such characteristics could include having a recent sexually transmitted infection; beliefs about a partner’s HIV status or viral suppression status; or other behavioural, biological or demographic indicators of HIV risk. Because the cascade analysis exhibited little sensitivity to the target group, we expect that additional details on the target group are unlikely to substantially change the proportional fall‐off of impact along the cascade of prevention, but the characteristics would be important for estimating the absolute magnitude of impact. Additionally, fall‐off along the prevention cascade could depend on the specific sub‐population or reason for PrEP initiation – an inter‐dependence that our analysis did not capture.

Our analysis provides insight into the most important steps in the cascade of prevention, as well as the overall attenuation of impact when the steps of the cascade are combined. Guided by conceptual frameworks for prevention cascades, such modelling can integrate emerging evidence in order to help guide priorities for implementation science and product development.

## 5 CONCLUSIONS

Implementation challenges along the prevention cascade compound to diminish the population‐level impact of PrEP. Even after accounting for the delay in long‐acting PrEP availability in terms of HIV infections that will occur prior to rollout, long‐acting PrEP nonetheless exhibited the best‐performing prevention cascade over a 20‐ or 30‐year time horizon. Our analysis projects that uptake will constitute the largest step‐down in impact along the “oral conventional” and “oral innovative” PrEP cascades. For long‐acting PrEP, our analysis suggests that the greatest limitations on impact will be caused by product availability timelines in the short term, and retention in the long term. To maximize the impact of long‐acting PrEP, ensuring timely product approval and rollout is essential in the short term. In the long term, implementation research should focus on retention and re‐engagement of users.

## COMPETING INTEREST

The authors declare that they have no competing interests.

## AUTHORS’ CONTRIBUTIONS

EM, KP and AB conceived of the study. All authors reviewed the literature and other data sources. AB coded the model, ran the simulations, performed the analyses and drafted the manuscript. All authors edited the manuscript and have read and approved the final manuscript.

## Supporting information


**Figure S1.** Percentage of infections averted along prevention cascades for different target groups receiving PrEP in western Kenya. Arrows show absolute percentage decrease relative to the previous step of the cascade. Cascades are shown for the target group of adults age 15 to 29 in the four counties with HIV prevalence exceeding 10% (top left), AGYW ages 15 to 24 in the four counties with HIV prevalence exceeding 10% (top right), higher‐risk men in all counties, including clients of sex workers and those at risk of having multiple sex partners or participation in transactional sex (bottom left), and higher‐risk women in all counties, including sex workers and those at risk of multiple sex partners or participation in transactional sex (bottom right). All scenarios use a 20‐year time horizon over 2020 to 2040.
