# Supplementary material for: Impact along the HIV pre‐exposure prophylaxis “cascade of prevention” in western Kenya: a mathematical modelling study
Source: J Int AIDS Soc. 2020 Jun 30;23(Suppl 3):e25527. doi: 10.1002/jia2.25527 (PMC7325506; doi:10.1002/jia2.25527)
Supplement: Supplementary file 1 — Figure S1. Percentage of infections averted along prevention cascades for different target groups receiving PrEP in western Kenya. Arrows show absolute percentage decrease relative to the previous step of the cascade. Cascades are shown for the target group of adults age 15 to 29 in the four counties with HIV prevalence exceeding 10% (top left), AGYW ages 15 to 24 in the four counties with HIV prevalence exceeding 10% (top right), higher‐risk men in all counties, including clients of sex workers and those at risk of having multiple sex partners or participation in transactional sex (bottom left), and higher‐risk women in all counties, including sex workers and those at risk of multiple sex partners or participation in transactional sex (bottom right). All scenarios use a 20‐year time horizon over 2020 to 2040. [file JIA2-23-e25527-s001.docx]

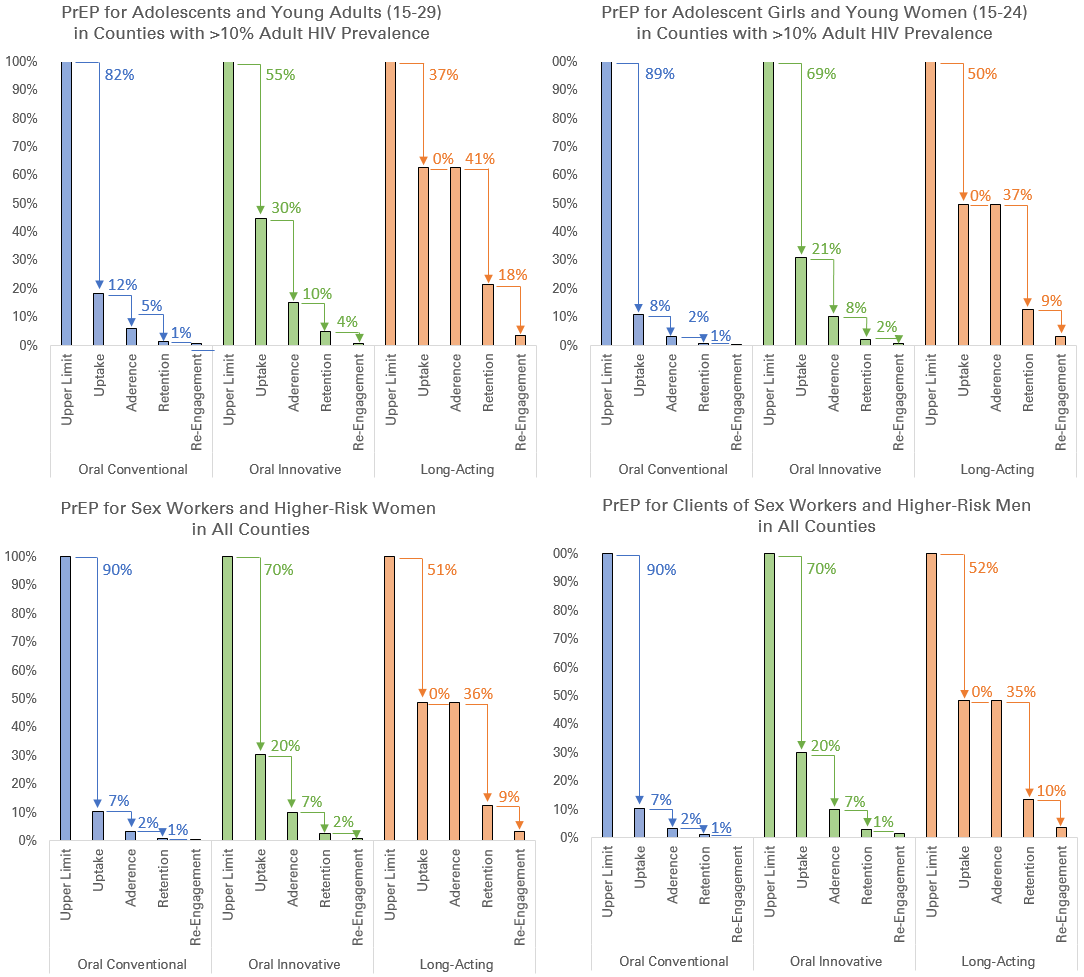


**Figure S1. Percentage of infections averted along prevention cascades for different target groups receiving PrEP in western Kenya**. Arrows show absolute percentage decrease relative to the previous step of the cascade. Cascades are shown for the target group of adults age 15-29 in the four counties with HIV prevalence exceeding 10% (top left), AGYW ages 15-24 in the four counties with HIV prevalence exceeding 10% (top right), higher-risk men in all counties, including clients of sex workers and those at risk of having multiple sex partners or participation in transactional sex (bottom left), and higher-risk women in all counties, including sex workers and those at risk of multiple sex partners or participation in transactional sex (bottom right). All scenarios use a 20-year time horizon over 2020-2040.
